# Sensor-Enabled Multi-Robot System for Automated Welding and In-Process Ultrasonic NDE

**DOI:** 10.3390/s21155077

**Published:** 2021-07-27

**Authors:** Momchil Vasilev, Charles N. MacLeod, Charalampos Loukas, Yashar Javadi, Randika K. W. Vithanage, David Lines, Ehsan Mohseni, Stephen Gareth Pierce, Anthony Gachagan

**Affiliations:** Centre for Ultrasonic Engineering (CUE), Department of Electronic & Electrical Engineering, University of Strathclyde, Glasgow G1 1XQ, UK; momchil.vasilev@strath.ac.uk (M.V.); charles.macleod@strath.ac.uk (C.N.M.); charalampos.loukas@strath.ac.uk (C.L.); randika.vithanage@strath.ac.uk (R.K.W.V.); david.lines@strath.ac.uk (D.L.); ehsan.mohseni@strath.ac.uk (E.M.); s.g.pierce@strath.ac.uk (S.G.P.); a.gachagan@strath.ac.uk (A.G.)

**Keywords:** non-destructive evaluation, robotic NDE, robotic welding, robotic control, in-process NDE, ultrasonic NDE, ultrasound

## Abstract

The growth of the automated welding sector and emerging technological requirements of Industry 4.0 have driven demand and research into intelligent sensor-enabled robotic systems. The higher production rates of automated welding have increased the need for fast, robotically deployed Non-Destructive Evaluation (NDE), replacing current time-consuming manually deployed inspection. This paper presents the development and deployment of a novel multi-robot system for automated welding and in-process NDE. Full external positional control is achieved in real time allowing for on-the-fly motion correction, based on multi-sensory input. The inspection capabilities of the system are demonstrated at three different stages of the manufacturing process: after all welding passes are complete; between individual welding passes; and during live-arc welding deposition. The specific advantages and challenges of each approach are outlined, and the defect detection capability is demonstrated through inspection of artificially induced defects. The developed system offers an early defect detection opportunity compared to current inspection methods, drastically reducing the delay between defect formation and discovery. This approach would enable in-process weld repair, leading to higher production efficiency, reduced rework rates and lower production costs.

## 1. Introduction

The automated welding industry has been valued at USD 5.5 billion in 2018 and is expected to double by 2026, reaching USD 10.8 billion [1] with industrial articulated robots predicted to replace current traditional column and boom systems and manual operations. This growth has been driven by key high-value manufacturing sectors including automotive, marine, nuclear, petrochemical and defence. Paired with the technological demands of Industry 4.0 [2], the need for the development of intelligent and flexible sensor-enabled robotic welding systems has become paramount.

The wide adoption of automated manufacturing systems has subsequently raised the demand for automatically deployed and adaptive Non-Destructive Evaluation (NDE) in order to keep up with the faster production lines, when compared to manual manufacturing processes [3]. Developments in automated NDE are driven by industrial demand for fast and reliable quality control in high-value and high-throughput applications. In general, automatic systems provide greater positional accuracy, repeatability and inspection rates when compared to human operators, therefore, resulting in faster inspection speeds and reduced manufacturing costs. The ever-improving capabilities of such systems, on the other hand, lead to an overall increase in asset integrity and lifecycle, resulting in further long-term savings. Safety is another key advantage of automated NDE systems, as they can be deployed in hazardous environments, dangerous conditions and sites where human access is limited or not possible [4,5], thus improving working conditions and reducing the risks of workplace injuries and harmful substance exposure [6].

Single-axis scanners offer the ability for axial or circumferential scans of pipes and are suitable for on-site inspection of assets such as oil and gas pipelines. Such scanners can be guided by a track, or can be free-rolling where a projected laser line is used by the operator to positionally align the scanner with the weld [7,8]. Mobile crawler systems offer a higher degree of positional flexibility through a two-axis differential drive and can magnetically attach to the surfaces of assets enabling vertical deployment [9]. In addition, their compact size makes them well suited for remote applications with constrained access [4]. One particular challenge with such crawlers is accurately tracking their position, which is achieved through a combination of drive encoders, accelerometers, machine vision and in often cases expensive external measurement systems [10]. Multirotor aerial vehicles can deliver visual [11], laser and, more recently, contact ultrasonic [12] sensors in remote NDE inspection scenarios, where a magnetic crawler could not be deployed. While umbilical/tether cables are used commonly with mobile crawlers, they pose a challenge for the manoeuvrability and range of aerial systems. As a result, the power source, driving electronics and data storage for NDE sensors need to be on board the multirotor and, therefore, must be designed according to its limited payload capabilities. These systems can typically position and orient sensors in four axes (X, Y, Z and yaw) with recently developed over-actuated UAVs aiming to overcome this in support of omnidirectional contact-based airborne inspection [13].

Fixed inspection systems offer a higher degree of positional accuracy, compared to mobile systems. Gantries and cartesian scanners operate in a planar or boxed work envelope and are suited for components with simple geometries. Articulated robotic arms, on the other hand, operate in a spherical work envelope and enable the precise delivery of sensors in six Degrees of Freedom (DoF) with pose repeatability of under ±0.05 mm and maximum linear velocities of 2 m/s [14]. They are widely used in industry thanks to their flexibility and reprogrammability, and their positional repeatability makes them suited for operations with well controlled conditions such as component dimensions, position and orientation. Seven DoF robots are also available, with the additional seventh axis in the form of a linear track or a rotational joint allowing a wider range of robot poses to reach the same end-effector position, enabling the inspection of more complex structures.

As specified in the international standards for ultrasonic NDE of welds [15,16,17], joints of metals with a thickness of 8 mm or above are to be tested with shear waves, inserted through contact angled wedges, where the induced ultrasonic beam must have a normal angle of incidence with the weld interface. The ultrasonic probe must be moved across the surface of the sample in a way that provides full coverage of the weld joint. Alternatively, a sweep of multiple beams across a range of angles can be induced via beamforming through a Phased Array Ultrasonic Transducer (PAUT) [18], forming a sectorial scan. Moreover, PAUT probes enable the acquisition of all transmit–receive pairs through Full Matrix Capture (FMC), which offers the advantage of retrospective beamforming and reconstruction of the weld area through the Total Focusing Method (TFM) [19,20].

NDE is a particular bottleneck when considering high-value automated welding, as it is traditionally performed days after manufacturing when the parts are allowed to cool down [15,16], to ensure cooling-related defects are found. As such, any defects that are detected in the welds and do not pass an acceptance criteria [17] would either require the part to be sent back for repairs or, in some cases, would lead to scrapping the component altogether. Apart from adding to the overall production process inefficiency, this problem also results in higher production costs and longer, less consistent lead times. This, paired with the fact that welds of thicker components, large bore pipes and Wire + Arc Additive Manufacture (WAAM) parts [21] require days and, in some cases, weeks to complete, increases the need for fast in-process NDE inspection. By integrating the inspection into the manufacturing process, an early indication of potential defects can be obtained, effectively addressing the production and cost inefficiencies by allowing for defects to be qualified and potentially repaired in-process.

Current state-of-the-art robotic NDE systems and automated welding systems rely on robot controllers for calculating the kinematics and executing the motion, which are usually programmed by users manually jogging the robot to individual positions through a teaching pendant. Furthermore, emerging sensors, such as optical laser profiles and cameras can be utilised and deployed to provide real-time path correction. However, the deployment of application-specific sensors is highly dependent on the commercially available software provided by industrial robot manufacturers and the supported communication protocols. Therefore, it would be particularly beneficial to bypass the internal motion planning of a robotic controller and to apply external real-time positional control, based on additional sensor inputs, effectively shifting the path planning and sensor integration to another controller. In particular, the Robot Sensor Interface (RSI) [22] communication protocol could be leveraged in order to provide such an external positional control capability.

RSI was developed by industrial robot manufacturer KUKA for influencing a pre-programmed motion path through sensor input in order to achieve an adaptive robotic behaviour. The protocol is based on an interpolation cycle, which executes in real-time intervals of 4 ms for KRC (KUKA Robot Controller) 4 controller-based robots, and 12 ms for legacy KRC 2-based robots. During this, an XML string with a special format is transmitted over a UDP (User Datagram Protocol) link between the robotic controller and an external sensor or system. In [3], RSI was used in conjunction with a force-torque sensor to maintain constant contact force between a composite wing component and an ultrasonic roller probe, effectively accounting for any discrepancies between the CAD model of the part and the as-built geometry. This method, however, required that the motion path is pre-set within a robotic program, making use of the built-in KUKA trajectory planning algorithm. In [23], a custom trajectory planning algorithm was developed and embedded on a KRC 4 controller through a real-time RSI configuration diagram. This gave the capability to dynamically set and update the target position over Ethernet and the layer of abstraction based on a C++ Dynamic Link Library (DLL), made it possible to utilise the toolbox in various programming environments, e.g., MATLAB, Python and LabVIEW. Although providing a fast response time, the toolbox did not have a provision for real-time motion correction based on sensory input and was fully reliant on the KRC for execution.

This paper presents the development of a sensor-enabled multi-robot system for automated welding and in-process ultrasonic NDE. Table 1 shows a comparison between this work and state-of-the-art commercial robotic NDE systems, i.e., Genesis Systems NSpect [24], TWI IntACom [25], Tecnatom RABIT [26], FRS Robotics URQC [27] and Spirit AeroSystems VIEWS [3]. A novel sensor-driven adaptive motion algorithm for the control of industrial robots has been developed. Full external positional control was achieved in real time allowing for on-the-fly motion correction, based on multi-sensory input. A novel multi-robot welding and NDE system was developed, allowing for the flexible manufacture of welded components and the research into, and deployment of, NDE techniques at the point of manufacture. Thus, the automatic high-temperature PAUT inspection of multi-pass welded samples at three distinct points of the welding manufacture has been made possible, for the first time: inspection of the hot as-welded components; interpass inspection, between welding pass deposition; and live-arc inspection, in parallel with the weld deposition. Through the insertion of artificially induced defects, it has been demonstrated that in-process ultrasonic inspection is capable of early defect detection, drastically reducing the delay between defect formation and discovery. Furthermore, the developed system has enabled the real-time control of the welding process through live-arc ultrasonic methods. Conventional PAUT and FMC are made possible through a high-speed ultrasonic phased array controller, allowing for the use of advanced image processing algorithms, producing results which cannot be achieved using conventional ultrasonics. The work presented herein has directly supported and enabled further research into in-process weld inspection, across sectors, with the aim of producing right-first-time welds. As a result, it is envisaged that future High Value Manufacturing (HVM) of welded components will have an increased component quality, process efficiency, and reduced rework rates, lead-time inconsistencies and overall costs.

## 2. Experimental System

### 2.1. Hardware

**Figure 1:**
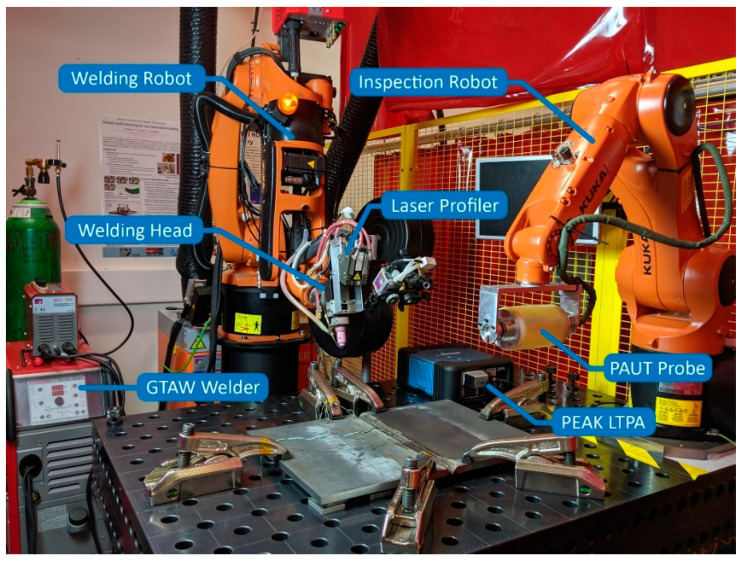
Sensor-enabled multi-robot welding and in-process NDE system.

The automated welding and NDE system depicted in Figure 1 is based around a National Instruments cRIO 9038 [28] real-time embedded controller. The cRIO features a real-time processor and a Field-Programmable Gate Array (FPGA) on board, which enables fast, real-time parallel computations. Eight expansion slots for additional Input/Output modules enable direct sensor connectivity in addition to the Ethernet, USB and other interfaces, featured on the cRIO. The expansion modules used were the NI 9476 Digital Output, NI 9263 Analogue Output, NI 9205 Analogue Input, NI 9505 DC Motor Drive and an NI 9214 Thermocouple module.

Automation was implemented through two 6 DoF industrial manipulators, controlled in real time through RSI over an Ethernet connection. A KUKA KR5 Arc HW with a KRC 2 controller was employed as the Welding Robot (WR), while a KUKA AGILUS KR3 with a KRC 4 controller was employed as the Inspection Robot (IR). The welding hardware comprised of a JÄCKLE/TPS ProTIG 350A AC/DC [29] welding power source and a TBi Industries water-cooled welding torch, mounted on the welding robot end effector. The welding arc was triggered through a 24 V digital signal connected to the power source, while the arc current was set through a 10 V differential analogue line. The power source featured process feedback in the form of measured arc current and arc voltage, also transmitted through differential analogue lines. A JÄCKLE/TPS 4-roll wire feeder, with an optical encoder was powered and controlled via the NI 9505. Its rotational speed was measured and controlled using Pulse Width Modulation (PWM) and was related appropriately to the desired control metric of linear wire feed rate. A Micro-Epsilon scanCONTROL 9030 [30] laser profiler was utilised for weld seam tracking and measurement, while an XIRIS XVC 1100 [31] high dynamic range weld monitoring camera provided visual feedback of the process.

**Figure 2:**
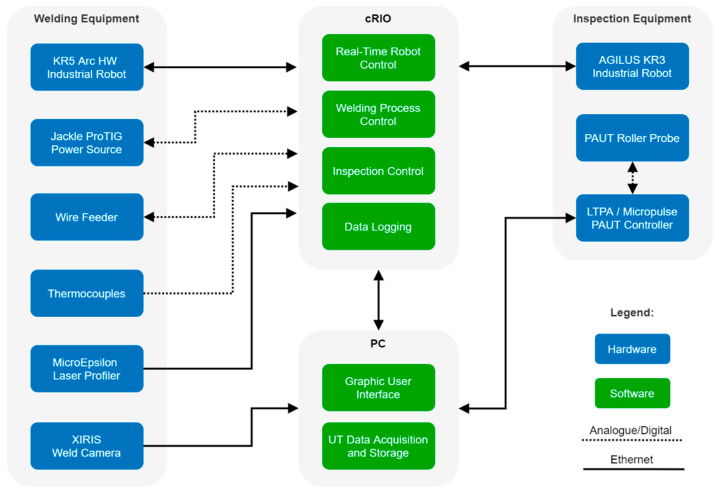
Sensor-enabled multi-robot welding and in-process NDE system architecture. Overall process control was implemented on the NI cRIO, while the GUI and PAUT acquisition and storage were executed on a host PC.

The workpiece temperature was measured through permanently attached thermocouples, which were used to maintain the workpiece within a desired interpass temperature range. The thermocouples were also utilised for monitoring the temperature gradient across the workpiece, which is a crucial requirement for temperature compensation of the ultrasonic images. A high-temperature PAUT roller probe was attached to the flange of the IR driven by a PEAK LTPA [32] low-noise ultrasonic phased array controller. The bandwidth and storage of the cRIO were only sufficient for inspection with conventional UT probes, therefore, the LTPA had to be directly connected to the host PC when using phased array probes. The bandwidth challenge could be addressed by substituting the cRIO with a high-performance NI PXI real-time controller. Finally, the Graphic User Interface (GUI) was deployed on the host PC, facilitating the user input, process monitoring and control. The high-level system architecture is shown in Figure 2, where the hardware components are represented by blue blocks, the software tasks are represented by green blocks and the communication links are shown as arrows.

### 2.2. Software

All software was developed in the cRIO native LabVIEW environment which enabled rapid prototyping, due to the wide range of supported communication protocols and software libraries. The software architecture was built using the JKI state machine [33] and parallel real-time Timed Loops, ensuring program flexibility while also providing reliable and fast response times. Three parallel state machines were responsible for executing the program sequence, controlling the Welding Robot (WR) and controlling the Inspection Robot (IR), respectively.

#### 2.2.1. Real-Time Robotic Control

The real-time robotic control strategy employed full external positional control of the robots. This was achieved through a correction-based RSI motion, meaning that the robot controller did not hold any pre-programmed path, and the robot end-effector position was updated on-the-fly through positional corrections. At every iteration of the interpolation cycle, the current position and timestamp of the internal clock are sent by the robot controller as an XML string. An XML string response is returned by the cRIO, mirroring the timestamp to keep the connection alive, and providing positional corrections in each axis, which determine where the end-effector will move to over the next interpolation cycle. There are two types of positional corrections—absolute, where the new position is given with respect of the robot base, and relative, where the new position is given with respect to the current position. For example, an absolute correction of 1 mm in the *X*-axis will move the end-effector to the absolute coordinate *X* = 1 mm, while the same relative correction will move the robotic end-effector by 1 mm in the positive *X*-axis direction irrespective of its current position. Relative corrections were chosen for this body of work as the smaller magnitude of corrections sent to the robot controller made them safer for use during the development and testing stage.

Welding and inspection robot paths are inputted by the user as individual points in a table through the GUI, where each row corresponds to a point in the path, while the columns hold the cartesian coordinates for each axis. Additional columns in the welding path table provide control over the process while approaching the target, i.e., an “Arc On” Boolean determines if the WR should be welding, and a “Log On” Boolean enables the data logging. More sophisticated data can also be included as additional columns, for example, to choose the welding parameters through a lookup table containing the settings for root, hot, filling and cap passes, therefore allowing the user to enter the parameters from a relevant Welding Procedure Specification (WPS) document alongside the robotic path. When considering simpler geometries such as a plate or pipe butt-weld, the robotic paths can be manually entered as individual point coordinates; for example, a straight-line weld would only require two points—the start and the end of the weld. For more complex geometries this can be generated by Computer Aided Manufacture (CAM) or robotic path planning software and imported into the software [34,35,36].

#### 2.2.2. Trajectory Planning

**Figure 3:**
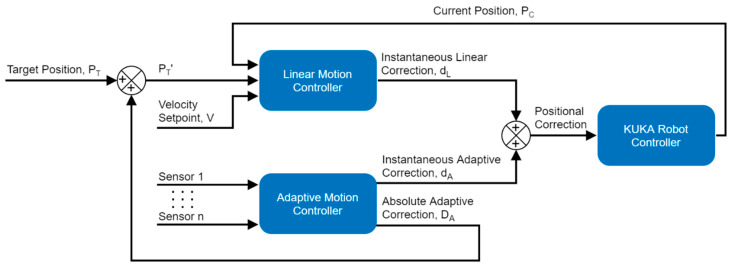
Trajectory planning and on-the-fly sensor-based motion correction algorithm.

An on-the-fly calculated trajectory planning algorithm running at the RSI interpolation cycle rate was implemented as demonstrated in Figure 3. A relative positional correction is sent to the KRC at each iteration of the interpolation cycle, consisting of a linear motion component d_L_ and an adaptive motion component d_A_. The Linear Motion Controller (LMC) is responsible for executing a straight-line trajectory between the current end-effector position P_C_ and a target position P_T_’. It is based on a linear acceleration–cruise–deceleration curve with the setpoint cruise speed V entered by the user. The Adaptive Motion Controller (AMC) generates an instantaneous adaptive correction d_A_ in response to the sensory input and process requirements. The absolute adaptive correction D_A_, which is the cumulative total correction that has been applied by the AMC, is summed to the current target position P_T_ taken from the robot path table to form P_T_’.

**Figure 4:**
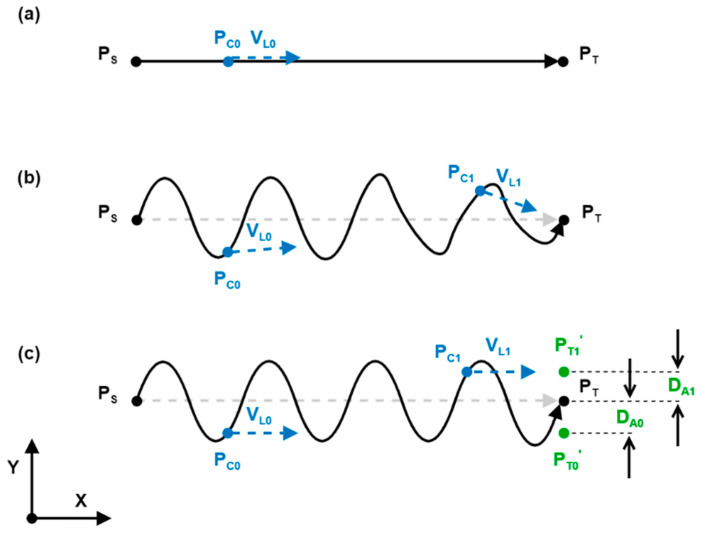
(**a**) Example linear motion generated by the LMC; (**b**) trajectory distortion introduced by instantaneous adaptive correction d_A_; (**c**) target point offsetting through absolute adaptive correction D_A_.

Figure 4a shows the operation of the LMC with an example linear trajectory along the *X*-axis between a starting point P_S_ and a termination point P_T_. The linear motion velocity vector V_L_ at an arbitrary point P_0_ along the path is always directed towards the target point P_T_ and is therefore parallel and coinciding with the P_S_P_T_ vector. Furthermore, as the P_S_P_T_ vector is aligned with the *X*-axis in Figure 4, the V_L_ vector only consists of an *X*-axis component. In Figure 4b, an example AMC output d_A_, consisting of a sinusoidal oscillation in the *Y*-axis, is summed with d_L_ before sending the positional correction to the KRC, resulting in a weaving motion between P_S_ and P_T_. However, as the linear motion vector V_L_ is always directed towards the target P_T_, a *Y*-axis component is introduced at all points that do not lie on the P_S_P_T_ vector, which results in a distorted trajectory. The effects of this distortion become stronger and more evident closer to P_T_ as illustrated by V_L0_ and V_L1_ in Figure 4b. In order to avoid the distortion in the LMC trajectory caused by the instantaneous correction d_A_, the absolute adaptive correction D_A_ is summed with P_T_ to give P_T_’. This offsetting of the target point ensures that the LMC-generated trajectory remains linear as shown in Figure 4c. As a result, a trade-off between target point accuracy and adaptive correction is inherently introduced in the system.

**Figure 5:**
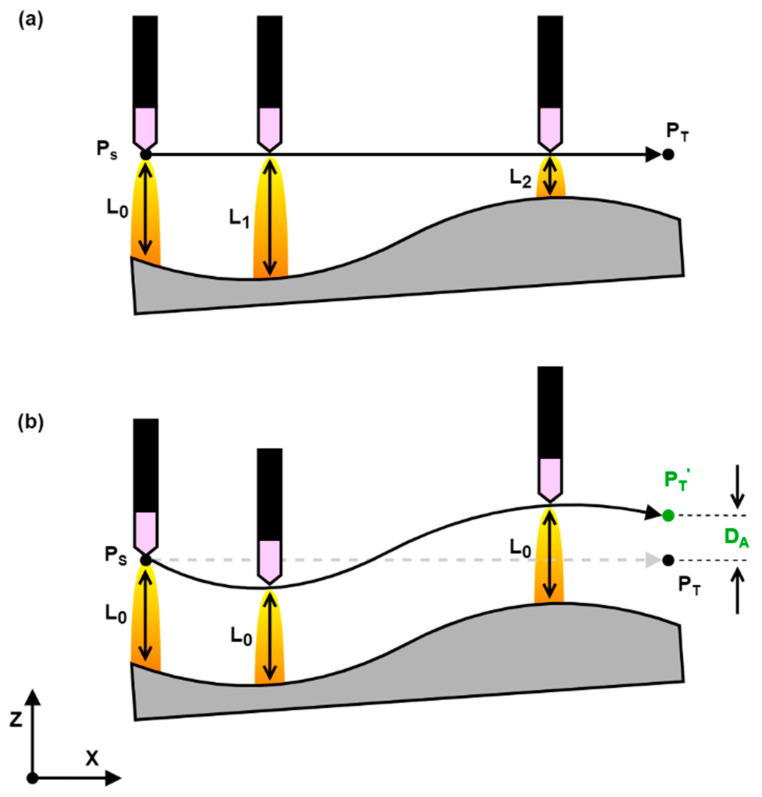
(**a**) Open-loop welding of a sample with an uneven surface through a linear trajectory; the welding torch to sample distance changes along the weld; (**b**) closed-loop welding of a sample with an uneven surface through an adaptive trajectory; on-the-fly adjustment of torch offset is achieved through the measured arc voltage; the welding torch to sample distance is constant along the weld; the end point P_T_ is shifted to P_T_’ as a result of the adaptive motion.

The demonstrated weaving motion is useful in various scenarios; for example, in welding, when mimicking the motion of manual welding techniques. Such a weaving motion is generally not achievable through a robotic teach pendant and requires path planning software. The software would normally create the path through a number of fundamental linear and circular motions, which would require a full trajectory recalculation if any of the parameters such as the travel speed, amplitude or frequency of weaving need to be modified. In contrast, as the weaving motion is calculated in real time, its parameters and driving function can be readily changed and updated on-the-fly. This approach can be applied to multiple axes at the same time and can be implemented with multiple sensors. For example, most modern automated welding power supplies offer the ability to monitor the arc current and arc voltage in real time, which can be utilised for process control. The measured arc voltage in the Gas Tungsten Arc Welding (GTAW) process is directly correlated to the distance between the welding torch and the workpiece, and as such is suitable for adaptive motion. When welding a workpiece that is assumed to be flat, but has surface height variations, the offset between the welding torch and the sample surface would vary along the weld as shown in Figure 5a, resulting in an inconsistent arc voltage and, therefore, inconsistent weld properties. The measured arc voltage was used as the control variable of a Proportional–Integral–Derivative (PID) control loop, the output of which was an instantaneous adaptive correction applied in the *Z*-axis. This allowed for Automatic Voltage Control (AVC), subsequently maintaining that the welding torch to workpiece distance is constant as illustrated in Figure 5b. The demonstrated approach can be applied for a variety of scenarios with equipment such as laser profilers, force-torque sensors and machine vision cameras among others.

#### 2.2.3. Welding Sequence

**Figure 6:**
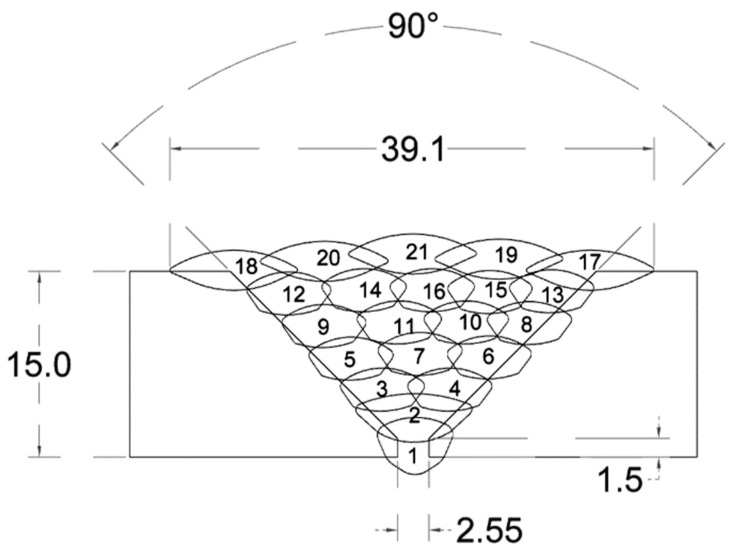
Multipass weld specification for 15 mm thick S275 steel bevelled with a 90° V-groove; a total of 21 passes are deposited over 7 layers; all linear dimensions are in millimetres.

All relevant process parameters and ultrasonic measurements were timestamped, positionally encoded by the robot position and logged in a binary format for subsequent analysis. Before any welding, the WR performed a calibration using the laser profiler in order to measure and locate the weld groove. This was performed only once, as the workpieces were fixed to the table using 6-point clamping and their location was not expected to shift with respect to the WR. In applications where an initial scan of the weld groove is not practical, or where the weld groove is expected to shift, the welding system has the capability to utilise the laser profiler output for real-time seam tracking, through the AMC. All multipass welding and inspection trials were performed on 15 mm thick S275 structural steel plates, bevelled to form a 90° V-groove. The plates were butt-welded by the WR over a total of 21 passes deposited over 7 layers, as shown in Figure 6.

## 3. Ultrasonic Inspection

The system was developed with the aim to perform ultrasonic inspections at three distinct points of the welding process: post-process, when all welding is completed; interpass in-process, between distinct welding passes; and live-arc in-process, in parallel with the weld deposition. Despite the distinct advantages and disadvantages of each approach, they would all fundamentally lead to early defect detection.

### 3.1. Post-Process UT

**Figure 7:**
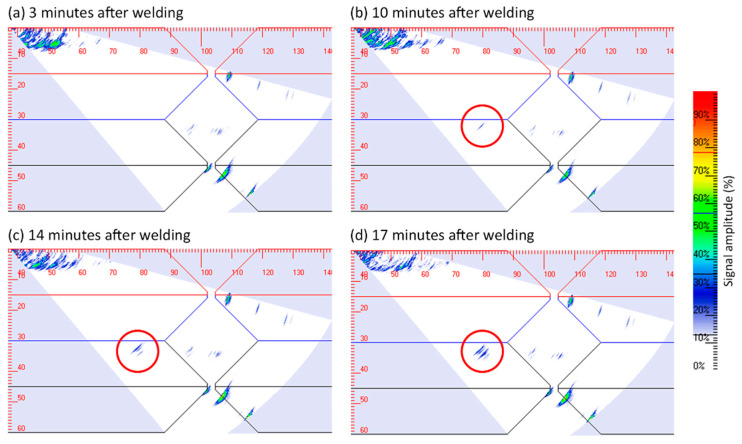
Continuous post-weld ultrasonic imaging of artificially induced hydrogen crack. The crack was initiated 10 min after all welding passes were deposited and its growth was observed in time. The location of the crack was in the HAZ adjacent to the weld cap toe.

The accuracy and positional repeatability of robots can be leveraged for post-process NDE by performing continuous repeated inspections of the as-built component. This allows for the development of any defects such as cold cracking to be monitored by comparing successive ultrasonic images. Due to the elevated sample temperature introduced by the welding process and any post-heat treatment, a high-temperature capable ultrasonic probe assembly was necessary. An Olympus 5L64-32 × 10-A32-P-2.5-HY array (5 MHz, 64 element, 0.5 mm element pitch, 10 mm element elevation) was used in conjunction with an SA32C-ULT-N55S-IHC angled wedge (suited for shear wave inspection in steel centred around 55°). The wedge is manufactured out of the material ULTEM and so is capable of short-term contact temperatures of up to 150 °C. High-temperature ultrasonic couplant was used between the transducer and wedge. Before touching down on each inspection position, the ultrasonic wedge was dipped in a custom-designed tray containing the same high-temperature ultrasonic couplant to ensure good acoustic propagation between probe and sample. Figure 7 shows the detection and growth monitoring of a hydrogen crack that was artificially induced in the Heat Affected Zone (HAZ), adjacent to the weld toe, through localised water quenching [37].

The elevated temperature of the sample after it is manufactured must be taken into account when performing NDE as the speed of sound in the material varies with temperature. As the sample cools down, this causes imaging anomalies in both amplitude and position. In [38], a Tungsten rod was introduced in the weld to form a static reflector of known size and location [39]. The weld was repeatedly inspected at regular time intervals for a period of 22 h, and the position and amplitude of the inserted reflector were extracted to form a thermal compensation curve. The sample temperature at the inspection location decreased from 164 °C at 2 min after welding to 28 °C at 75 min after welding. As a result, the reflected amplitude increased significantly from 25% to 62% of full screen height, and the defect indication’s position shifted by 3 mm on the reconstructed sector scan image. These data were utilised to correct the position and amplitude of an artificially induced crack. The crack initiation was successfully detected 22 min after the weld completion, and it was observed to be growing over a total of 90 min.

### 3.2. Interpass In-Process UT

**Figure 8:**
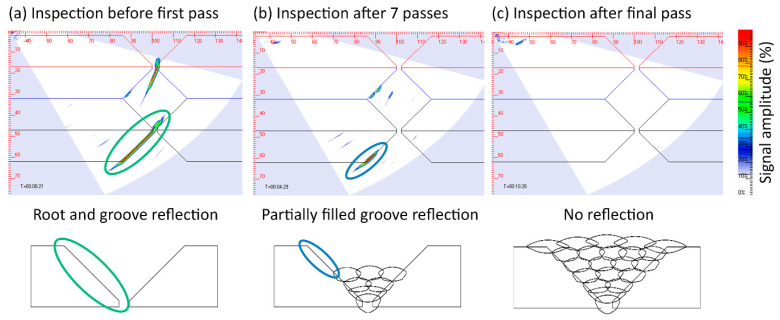
Ultrasonic sectorial scan of 90° V-groove multipass weld; (**a**) before welding the groove edge is detected as a reflector (green marker); (**b**) after 7 passes are deposited, the size of the groove edge indication is reduced (blue marker); (**c**) after all welding passes are deposited, the groove edge is no longer detected.

Interpass ultrasonic NDE allows for the detection of weld flaws through inspection between individual welding passes or layers and provides an opportunity for in-process repair, as only a small amount of material would need to be removed in order to excavate and repair the defects. This is particularly advantageous for the manufacture of components that are typically challenging to repair after all welding passes have been deposited, e.g., thick multipass welds and WAAM parts. A key challenge of interpass welding inspection is the complex sample geometry which changes as the weld is deposited and therefore differs from the as-built geometry [40]. Figure 8 shows that the unwelded portion of the V-groove in a multipass weld causes a number of reflections and artefacts in the ultrasonic images, as demonstrated at three distinct stages of the sample manufacture. As the weld is deposited, the sample geometry reflections change in shape and size, until they disappear upon completion of the weld joint. Hence, appropriate signal processing and masking are required to effectively remove the false positive indications from the sample geometry.

**Figure 9:**
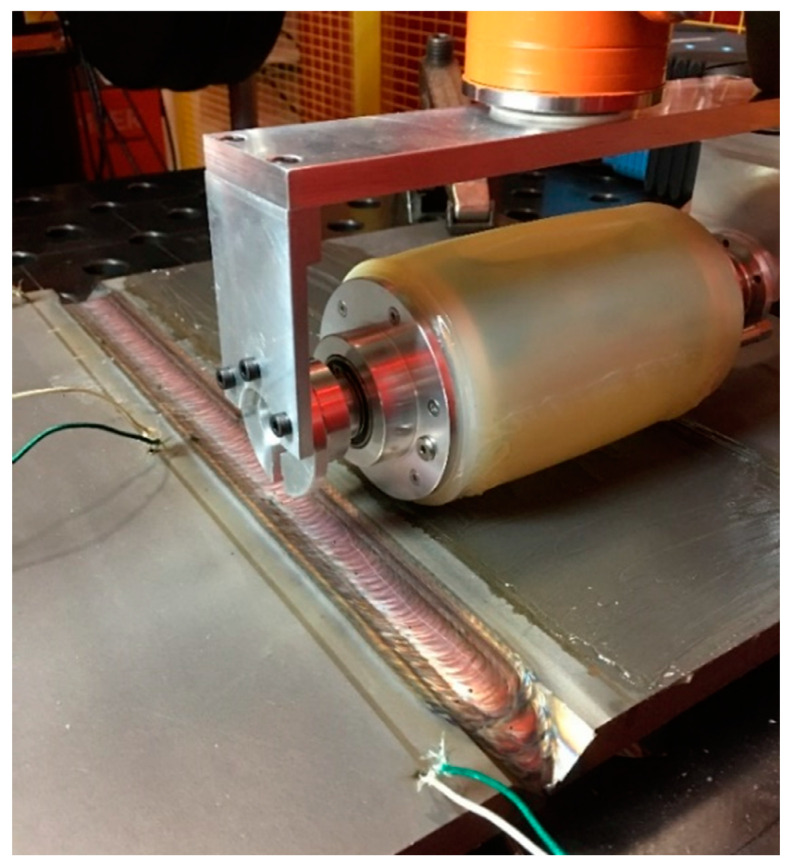
Interpass in-process UT inspection with a novel high-temperature PAUT roller probe.

**Figure 10:**
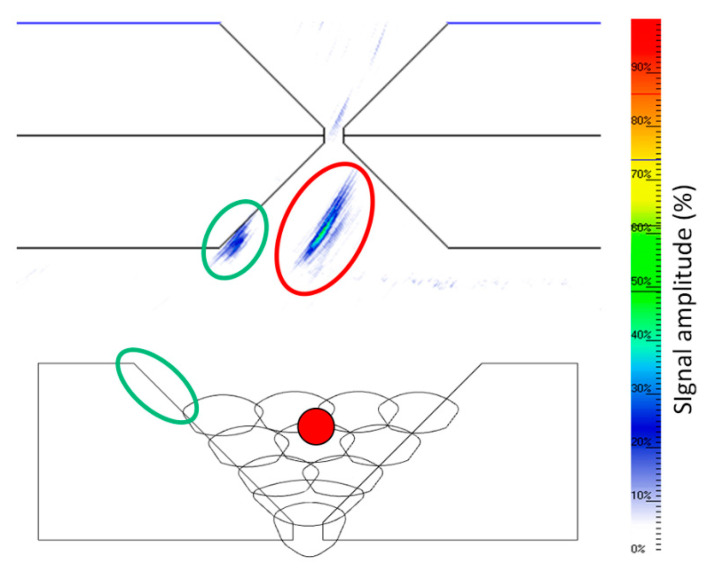
Interpass ultrasonic image of artificially induced defect (Tungsten rod with 2.4 mm diameter) (red marker) with a false positive indication from the unwelded groove edge (green marker).

The high interpass temperatures required to maintain the weld integrity (typically up to 250 °C) have driven research into the development of a novel, high-temperature capable PAUT probe [41]. The probe features a 5 MHz, linear 64-element PAUT transducer immersed in water and enclosed in a moulded high-temperature silicone rubber tyre, capable of operating at temperatures up to 350 °C. Coupling between the probe and the sample was achieved through a constant compressional force and high-temperature gel couplant as demonstrated in Figure 9. The novel probe has allowed for the interpass detection of artificially induced defects inside a partially filled multipass weld such as the one shown in Figure 10, where a Tungsten rod with a diameter of 2.4 mm and length of 30 mm was included in the weld.

As a result of the moving heat source in welding, thermal gradients in both the direction of welding and perpendicular to the direction of travel are introduced in the workpiece, ultimately resulting in ultrasonic image distortion. Furthermore, the dynamic nature of multipass welding essentially results in a different thermal gradient after each welding pass. An in-process thermal compensation procedure was proposed in [42] involving the parallel manufacture of a second, identical sample with an embedded Tungsten pipe, serving as an in-process calibration block. The reflection from the known in size and location pipe was used to calibrate for the effects of the temperature gradients and it was demonstrated that the approach provided more accurate results, compared to a traditional calibration on a sample with a side drilled hole at a uniform temperature. For the most accurate calibration and thermal compensation results, however, the sample temperature would need to be precisely known through a combination of measurement and weld modelling [43]. It is important to also note that interpass inspection could increase the component manufacture duration, as it is deployed sequentially with the welding deposition. In addition, increasing the interval between welding passes could lead to excessive sample cooling and the loss of interpass temperature. Therefore, the UT acquisition and image processing speed must be taken into account when considering the deployment of interpass NDE for welding applications.

### 3.3. Live-Arc In-Process UT

**Figure 11:**
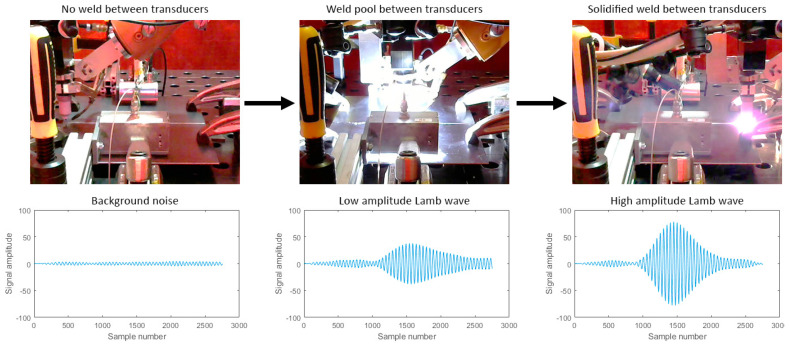
Live-arc in-process weld UT with non-contact Lamb waves.

**Figure 12:**
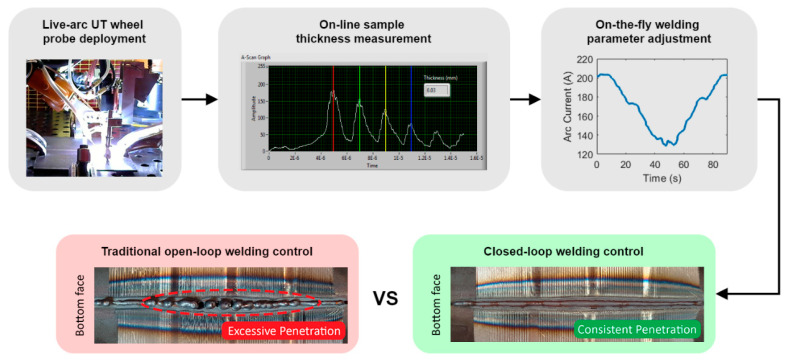
On-the-fly adaptive welding control through live-arc UT sample measurement.

In-process UT deployed during the welding deposition offers rapid feedback for the welding process and allows not only measurement, but also control of the welding process. In [44], a pair of air-coupled ultrasonic transducers were used to induce guided Lamb waves through a section of 3 mm thick plate butt joint while it was deposited. Figure 11 shows that the solidification of the weld was monitored in real time through live-arc in-process UT. This method has shown promise as the rate of change of the received Lamb waves’ amplitude was found to be correlated to the weld penetration depth. In [45], a split-crystal ultrasonic wheel probe was attached to the welding torch and was utilised for thickness measurement of samples with a varying loss of wall thickness, as shown in Figure 12. The measured thickness was used to control and adapt the welding arc current, torch travel speed and wire feed rate on-the-fly. It was demonstrated that the approach provided sufficiently low latency and high accuracy for real-time welding process control and, as a result, provided a better performance of welding samples with thickness variations, compared to a traditional open-loop automated welding system.

**Figure 13:**
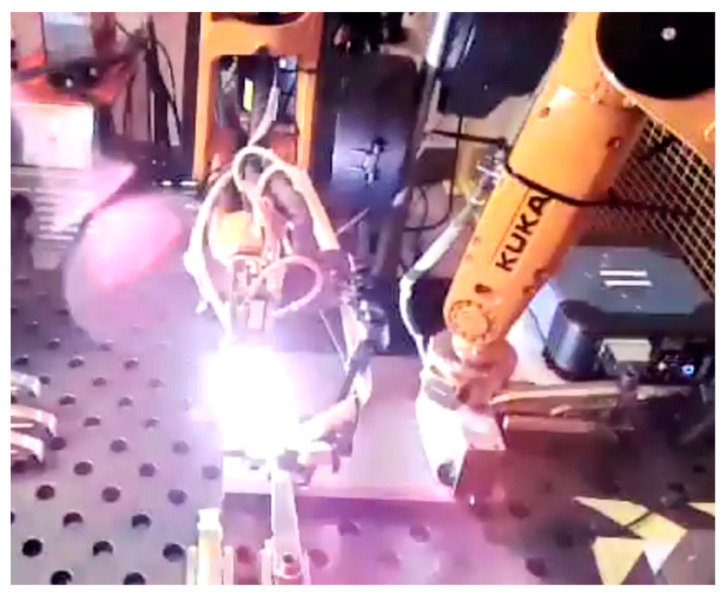
Live-arc PAUT inspection experiment.

Current work in the University of Strathclyde is focused on addressing the challenges associated with deploying PAUT probes during the weld deposition (Figure 13). The next generation of PAUT probes will be dry coupled, which would remove the risk of unwanted weld contamination by the ultrasonic gel that can cause porosity [37] and would reduce the variation in coupling between the probe and the workpiece.

## 4. Conclusions and Future Work

A novel sensor-enabled robotic system for automated welding and ultrasonic inspection was developed and evaluated. The system architecture was based around an NI cRIO real-time embedded controller which enabled real-time communication, data acquisition and control. A real-time external robotic control strategy for adaptive behaviour was developed, allowing for on-the-fly sensor-based trajectory corrections. The inspection capabilities of the system were demonstrated in three different scenarios:

1. Post-process continuous UT—the initiation and growth of cold crack defects was observed and measured through a continuous inspection at regular intervals after the multipass weld was completely filled.
2. Interpass in-process UT—the challenges due to the complex sample geometry of the unfilled weld groove were demonstrated and the inspection results showed the defect detection capabilities through artificially induced defects.
3. Live-arc in-process UT—the deployment and application of three different ultrasonic sensors during live-arc welding deposition was outlined and the challenges and results were discussed.

Current work on masking the bevel edge reflections will remove false positives arising from the unfilled weld groove and thermal gradient compensation would enable the accurate locating and sizing of weld defects. Future developments of the PAUT probe will allow for a completely dry coupled inspection, eliminating the coupling and contamination challenges posed by the ultrasonic couplant. It is envisaged that future welding and live-arc in-process systems would possess the capability for automatic in-process defect detection, which would in turn significantly reduce the delay between the development and detection of a defect, offering the potential for in-process weld repair.

## Figures and Tables

**Table 1:** Comparison between state-of-the-art commercial robotic NDE systems and this work.

	NSpect	IntACom	RABIT	URQC	VIEWS	This Work
Automated robotic NDE	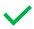	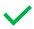	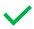	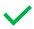	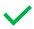	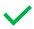
Adaptive motion	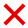	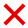	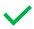	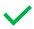	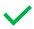	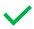
FMC capture	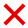	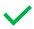	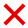	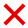	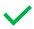	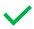
Real-time trajectory control	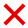	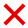	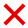	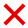	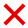	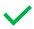
Sensor integration independent from robot controller	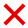	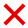	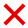	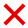	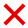	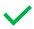
NDE integrated with manufacture	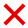	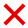	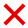	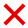	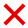	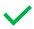
High temperature inspection	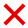	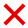	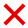	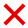	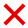	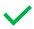

Where 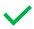 denotes yes and 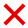 denotes no.

## Data Availability

Not applicable.
